# Triglyceride-glucose index is associated with cerebral small vessel disease-related motor impairment in Parkinson’s disease: a mediation analysis

**DOI:** 10.3389/fnagi.2026.1902923

**Published:** 2026-08-10

**Authors:** Meimei Zhang, Wei Li, Yumei Zhang, Tao Feng

**Affiliations:** 1 Department of Neurology II, Shaanxi Provincial People’s Hospital, Xi’an, Shaanxi, China; 2 China National Clinical Research Center for Neurological Diseases (NCRC-ND), Beijing, China; 3 Department of Rehabilitation, Beijing Tiantan Hospital, Capital Medical University, Beijing, China; 4 Department of Neurology, Beijing Tiantan Hospital, Capital Medical University, Beijing, China

**Keywords:** cerebral small vessel disease, mediation analysis, motor impairment, Parkinson’s disease, triglyceride-glucose index

## Abstract

**Background:**

Cerebral small vessel disease (CSVD) may worsen motor and cognitive impairment in Parkinson’s disease (PD), yet whether peripheral metabolic inflammation mediates this link remains unclear. The triglyceride-glucose (TyG) index, a surrogate for insulin resistance, has been associated with CSVD and neurodegeneration. This study examined whether the TyG index statistically accounts for the association between CSVD burden and motor or cognitive deficits in PD.

**Methods:**

This retrospective cross-sectional study enrolled 362 PD patients. The 4-point scale assessed CSVD burden. Movement Disorder Society Unified Parkinson’s Disease Rating Scale part III (MDS-UPDRS III) and Montreal Cognitive Assessment (MoCA) evaluated motor and cognitive function, separately. Spearman correlation and mediation analyses were performed, adjusting for age, sex, education, hypertension, diabetes, and daily dose equivalent of levodopa. Sensitivity analyses were conducted to assess the robustness and specificity of the findings.

**Results:**

CSVD burden was positively associated with MDS-UPDRS III (total effect = 2.309, 95% CI [0.463, 4.155, *p* = 0.014]) and negatively associated with TyG index (*a* = −0.067, *p* = 0.025). TyG index was negatively associated with MDS-UPDRS III (*b* = −6.561, *p* < 0.001). The indirect effect was 0.441 (95% CI [0.048, 0.958]), accounting for 19.1% of the total effect. This mediation was specific to TyG, as four alternative inflammatory indices showed no significant effects. Additional analyses adjusting for disease duration and Hoehn-Yahr staging confirmed robustness [indirect effect = 0.288, 95% CI (0.019, 0.687)]. Neither fasting triglycerides nor glucose alone mediated the effect. No mediation was found for cognition.

**Conclusion:**

The TyG index statistically accounts for part of the association between CSVD and motor function in PD via a negative-to-negative pathway, suggesting a hypothesis-generating metabolic target that warrants prospective investigation.

## 1 Introduction

Parkinson’s disease (PD) is the second most common neurodegenerative disorder, predominantly affecting individuals over the age of 65 (Ma et al., 2025b) and projected to pose a growing public health challenge for patients, caregivers, and society by 2050 (Dongning et al., 2025). Progressive motor and cognitive impairment are the prominent clinical features of PD, yet the severity and progression rate vary considerably among individuals. Accumulating evidence suggests that cerebral small vessel disease (CSVD), a common age-related vascular pathology, may contribute to this heterogeneity. PD patients with a higher CSVD burden always exhibit severe motor and cognitive impairment, especially gait and postural instability, along with rapid cognitive decline (Chen et al., 2020; Chen et al., 2021; Qiu et al., 2024). However, the mechanistic pathways linking CSVD to motor and cognitive impairment in PD remain poorly understood.

Peripheral inflammation and insulin resistance have been proposed to correlate with CSVD and neurodegeneration (Williams et al., 2022; Nowell et al., 2023; Van der Taelen et al., 2025; Su et al., 2026), suggesting that these factors may mediate vascular-neural interactions. The triglyceride-glucose (TyG) index, calculated from fasting triglyceride and glucose levels, serves as a validated surrogate of insulin resistance. Previous studies have demonstrated that a higher TyG index is associated with a greater burden of CSVD in both healthy individuals and patients with type 2 diabetes (Nam et al., 2020; Teng et al., 2022). However, paradoxical findings have emerged in patients with PD. Recent studies have reported a negative association between the TyG index and cognitive impairment (Cao et al., 2025; Cheng et al., 2025). Meanwhile, our earlier work demonstrated that lower serum triglyceride levels (consequently lower TyG index) were linked to more severe motor impairment (Zhang et al., 2022). This reversal may indicate a disease-specific metabolic shift in PD, in which a low TyG index signals disease-related malnutrition or energy depletion rather than metabolic health.

Despite these parallel lines of evidence, no study has directly examined whether the TyG index mediates the effect of CSVD on motor and cognitive impairment in PD. Traditional bivariate correlations cannot clearly determine whether CSVD exerts a detrimental effect through modifying metabolic-inflammatory status. We therefore performed a formal mediation analysis to test whether the TyG index statistically accounts for the relationship between CSVD burden and motor dysfunction in PD patients. As a secondary aim, we explored whether this pathway extends to cognitive impairment to assess domain specificity.

## 2 Materials and methods

### 2.1 Study design and participants

A retrospective cross-sectional study was conducted using data from patients hospitalized at the Department of Neurology of Beijing Tiantan Hospital, Capital Medical University, between January 2019 and September 2022. Sample size was not pre-calculated due to retrospective consecutive enrollment. Enrolled patients met the following inclusion criteria: 1) a clinical diagnosis of definite PD according to the Movement Disorder Society (MDS) criteria; 2) completion of relevant clinical, laboratory, and imaging examinations. Exclusion criteria include 1) a diagnosis of Parkinsonism or Parkinson-plus syndromes; 2) other neurological conditions that could lead to dementia, a history of stroke, brain injury, severe cardiovascular, liver, or kidney disorders, immune system diseases, or malignant tumors. This study was conducted in accordance with the Declaration of Helsinki and approved by the Ethics Committee of Beijing Tiantan Hospital.

### 2.2 Acquisition of laboratory data

Blood samples were collected in the early morning after at least 8 h of fasting. Peripheral blood was analyzed for neutrophil count, lymphocyte count, triglycerides, high-density lipoprotein cholesterol (HDL-C), and fasting glucose. The triglycerides and fasting glucose were originally measured in mmol/L and converted to mg/dL using standard conversion factors (triglycerides: × 88.57; glucose: × 18.02) before calculating the TyG index. The TyG index was calculated as ln [fasting triglycerides (mg/dl) × fasting glucose (mg/dl)/2]. Additional inflammatory indicators were calculated: SII (neutrophils×platelets/lymphocytes), SIRI (neutrophils×monocytes/lymphocytes), neutrophil-to-lymphocyte ratio (NLR), and neutrophil-to-HDL-C ratio (NHR).

### 2.3 Clinical assessments

Upon admission, demographic information and medical history, including age, sex, height, weight, years of education, disease duration, hypertension, and diabetes history, were collected. Body mass index (BMI) was calculated as weight in kilograms divided by height in meters squared. Hoehn-Yahr (H-Y) staging was used to assess the disease severity. Motor symptoms were evaluated with the Movement Disorder Society Unified Parkinson’s Disease Rating Scale part III (MDS-UPDRS III). Cognitive function was measured with the Montreal Cognitive Assessment (MoCA). MoCA scores were adjusted for education according to standard guidelines: one point was added for participants with ≤ 12 years of education. The daily dose equivalent of levodopa (LEDD) was calculated using standard protocols. The evaluations were conducted in the “ON state” (1 to 2 h after medication intake).

### 2.4 MRI imaging data and CSVD burden evaluation

MRI data were acquired from a 3 T scanner (Philips Ingenia) at Beijing Tiantan Hospital using T1-weighted, T2-weighted, fluid-attenuated inversion recovery, and susceptibility-weighted imaging (SWI) sequences. Due to the retrospective nature of this study, detailed acquisition parameters such as repetition time and echo time were not consistently recorded in the original imaging database. All scans followed the institutional standard clinical protocol for CSVD evaluation, which is consistent with the STRIVE criteria (Duering et al., 2023). Slice thickness was 5 mm for conventional sequences and 1–2 mm for T1-weighted and SWI sequences, in accordance with routine clinical practice. All image readers received professional training and were blinded to each other’s assessment as well as to patients’ demographic and clinical data. Two readers independently interpreted the same images, and a senior radiologist conducted a final review to ensure the accuracy and consistency of the imaging findings. Inter-rater reliability was assessed using Cohen’s kappa, yielding values of 0.824 for white matter hyperintensities, 0.788 for lacunes, 0.794 for enlarged perivascular spaces, and 0.805 for microbleeds, indicating good to excellent agreement.

The severity of CSVD was evaluated using the 4-point burden scale. Points were assigned based on the following MRI features: periventricular hyperintensities (Fazekas grade 3) or deep white matter hyperintensities (Fazekas grade 2–3) received one point; moderate to severe enlarged perivascular spaces in the basal ganglia received one point; one or more lacunes received one point; and one or more microbleeds received one point(Staals et al., 2014). The assessment of white matter hyperintensities, enlarged perivascular spaces, and microbleeds followed previously established standards in the literature (Fazekas et al., 1993; Gregoire et al., 2009; Doubal et al., 2010; Wardlaw et al., 2013).

### 2.5 Variables definition

Exposure variable: CSVD burden.

Mediator: TyG index.

Primary outcome: MDS-UPDRS III.

Secondary outcome: MoCA.

Confounders: age, sex, years of education, hypertension, diabetes, and LEDD.

Additional covariates (adjusted in sensitivity analyses): BMI, disease duration, and H-Y staging.

### 2.6 Statistical analyses

All statistical analyses were performed using SPSS 26.0. The normality of the data was checked by the Shapiro–Wilk test. Continuous variables were presented as means ± standard deviation (SD) or medians with interquartile ranges (IQRs), depending on the distribution characteristics. Categorical variables were described using counts and percentages. Differences between low and high CSVD burden groups (dichotomized at the median) were evaluated using Student’s t-test or Mann–Whitney U test. Spearman correlation analysis was employed to examine associations among CSVD burden, TyG index, motor and cognitive function.

Primary mediation analysis was conducted using Hayes’ PROCESS macro (version 4.2, Model 4) for SPSS, with 5,000 bootstrap resamples to compute 95% confidence intervals (CIs) and standard errors (SEs). CSVD burden was modeled as a continuous variable, a choice justified by the non-significant quadratic terms in regression models for TyG index (*p* = 0.077) and MDS-UPDRS III (*p* = 0.225), which supported the assumption of linearity. Model assumptions for the mediation analysis were assessed. Residuals were approximately normal, no heteroscedasticity was evident, and all variance inflation factor values remained below 2, indicating no collinearity. The PROCESS employs listwise deletion for cases with incomplete data on any variable in the model. Of the 371 patients initially assessed, 7 were excluded for missing data and 2 for a history of stroke, yielding a final sample of 362. CSVD burden was specified as the independent variable (X), TyG index as the mediator (M), and MDS-UPDRS III as the dependent variable (Y). Age, sex, years of education, hypertension, diabetes, and LEDD were included as covariates based on established literature and clinical plausibility (Chen et al., 2021). An indirect effect was considered statistically significant if its bootstrap CI excluded zero. Total, direct, and indirect effects were reported with their 95% CIs.

Sensitivity analyses were performed to assess the robustness and specificity of the mediation findings. First, we repeated the mediation model using four alternative peripheral inflammatory indicators previously reported to be associated with CSVD and motor symptoms in PD (Xiao et al., 2024; Yang et al., 2025), including SII, SIRI, NLR, and NHR. Second, CSVD burden was dichotomized to examine whether the mediation effect remained with a categorical exposure. Third, BMI was additionally included as a covariate to control for potential confounding by overall adiposity. Fourth, disease duration and H-Y staging were additionally included as covariates to control for disease severity. Fifth, to decompose the TyG index, separate mediation models were performed using fasting triglycerides and glucose as independent mediators in both forward (CSVD burden→M → MDS-UPDRS III) and reverse (MDS-UPDRS III → M → CSVD burden) directions. All sensitivity analyses used the same bootstrap procedure and covariate adjustment as the primary analysis.

Secondary mediation analysis, with MoCA score as the dependent variable, was also performed as a prespecified exploratory analysis to determine whether the same mediation pathway extended to cognitive function. The same model specifications and covariate adjustments were applied.

Potential sources of bias were addressed through standardized data collection protocols, independent image interpretation by two trained readers, and adjustment for prespecified confounders in the mediation model.

All statistical tests were two-sided, and a *p*-value < 0.05 was considered statistically significant.

## 3 Results

### 3.1 Characteristics of the PD patients

**Figure 1:**
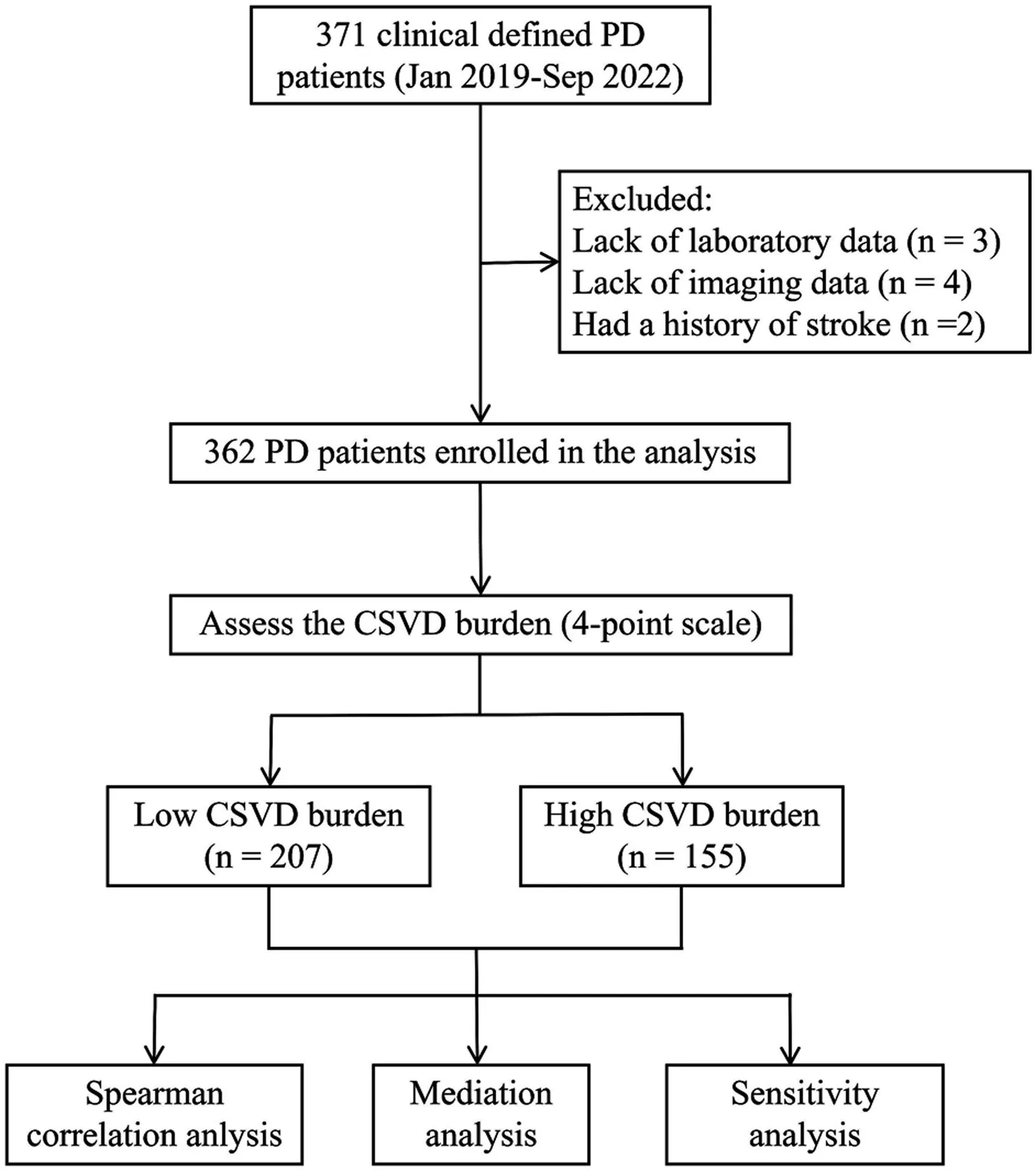
Flowchart of patient inclusion. CSVD, cerebral small vessel disease burden; PD, Parkinson’s disease.

After excluding 7 patients with missing data and 2 with a history of stroke, a total of 362 PD patients were enrolled in the final analysis (Figure 1). Table 1 summarizes the demographic, medical history, clinical, laboratory indicators, and CSVD burden characteristics of the participants. The median age of the participants was 64.00 years, and the median H-Y staging was 3.00. PD patients were divided into two groups according to the median CSVD burden (low burden group: CSVD burden ≤ 1; high burden group: CSVD burden > 1). Compared with the low burden group, the high burden group was older, had lower educational attainment, a higher prevalence of hypertension, lower triglyceride levels, and higher SIRI and NLR levels (*p* < 0.01, *p* < 0.05; Table 1). The distribution of each CSVD imaging marker was presented in Supplementary Table S1.

**Table 1:** Characteristics of PD patients between low and high CSVD burdens.

Characteristics	Total (*n* = 362)	Low burden (*n* = 207)	High burden (*n* = 155)	*p*
Demographic				
Age, years, median (IQR)	64.00 (58.00, 69.00)	63.00 (57.00, 68.00)	66.00 (60.00, 72.00)	**<0.001**
Male (%)	212, 58.56%	114, 55.07%	98, 63.23%	0.119
Years of education, median (IQR)	10.00 (9.00, 12.00)	12.00 (9.00, 12.00)	9.00 (8.00, 12.00)	**0.027**
BMI, kg/m^2^, median (IQR)	24.21 (21.99, 26.47)	24.01 (21.88, 26.67)	24.22 (22.15, 26.22)	0.766
Medical history				
Hypertension, *n* (%)	114, 31.49%	49, 23.67%	65, 41.94%	**<0.001**
Diabetes, *n* (%)	47, 12.98%	21, 10.14%	26, 16.77%	0.063
Clinical				
Disease duration, years, median (IQR)	6.00 (4.00, 10.00)	6.00 (4.00, 8.00)	7.00 (4.00, 10.00)	0.055
H-Y staging, median (IQR)	3.00 (2.00, 3.00)	3.00 (2.00, 3.00)	3.00 (2.00, 4.00)	0.210
LEDD, mg/day, median (IQR)	500.00 (325.00, 750.00)	500.00 (300.00, 725.00)	580.00 (375.00, 775.00)	0.064
MDS-UPDRS III, median (IQR)	39.00 (28.00, 51.00)	38.00 (28.00, 49.00)	40.00 (28.25, 53.75)	0.090
MoCA, median (IQR)	23.00 (18.00, 26.00)	23.00 (19.00, 26.00)	22.00 (16.75, 26.00)	0.140
Laboratory indicators				
Triglyceride, mmol/L, median (IQR)	1.04 (0.75, 1.48)	1.07 (0.77, 1.54)	0.96 (0.72, 1.38)	**0.040**
Fasting glucose, mmol/L, median (IQR)	4.53 (4.25, 5.03)	4.50 (4.22, 4.96)	4.58 (4.31, 5.11)	0.095
TyG index, median (IQR)	8.26(7.91, 8.66)	8.29 (7.94, 8.69)	8.17 (7.88, 8.59)	0.150
SII, median (IQR)	21.45 (14.19, 34.04)	21.44 (13.98, 32.87)	21.75 (15.40, 36.05)	0.138
SIRI, median (IQR)	0.68 (0.48, 1.01)	0.62 (0.46, 0.93)	0.73 (0.54, 1.16)	**0.001**
NLR, median (IQR)	0.11 (0.08, 0.15)	0.10 (0.08, 0.14)	0.11 (0.08, 0.17)	**0.040**
NHR, median (IQR)	2.68 (2.02, 3.59)	2.67 (1.99, 3.54)	2.71 (2.05, 3.83)	0.308
CSVD burden	1.00 (1.00, 2.00)	/	/	/

Normally and nonnormally distributed continuous variables are expressed as mean ± SD and median (IQR), respectively; categorical variables are expressed as number (percentage %). Bold values indicate statistically significant differences.

CSVD, cerebral small vessel disease burden; BMI, body mass index; LEDD, levodopa equivalent daily dosage; H-Y staging, Hoehn–Yahr staging; MDS-UPDRS III, Movement disorder society-unified Parkinson’s disease rating scale part III; MoCA, Montreal Cognitive Assessment; TyG index, triglyceride-glucose index; SII, neutrophils×platelets/lymphocytes; SIRI, neutrophils×monocytes/lymphocytes; NLR, neutrophil-to-lymphocyte ratio; NHR, neutrophil-to-HDL-C ratio. Statistically significant results are shown in bold.

### 3.2 Spearman correlation analysis

**Figure 2:**
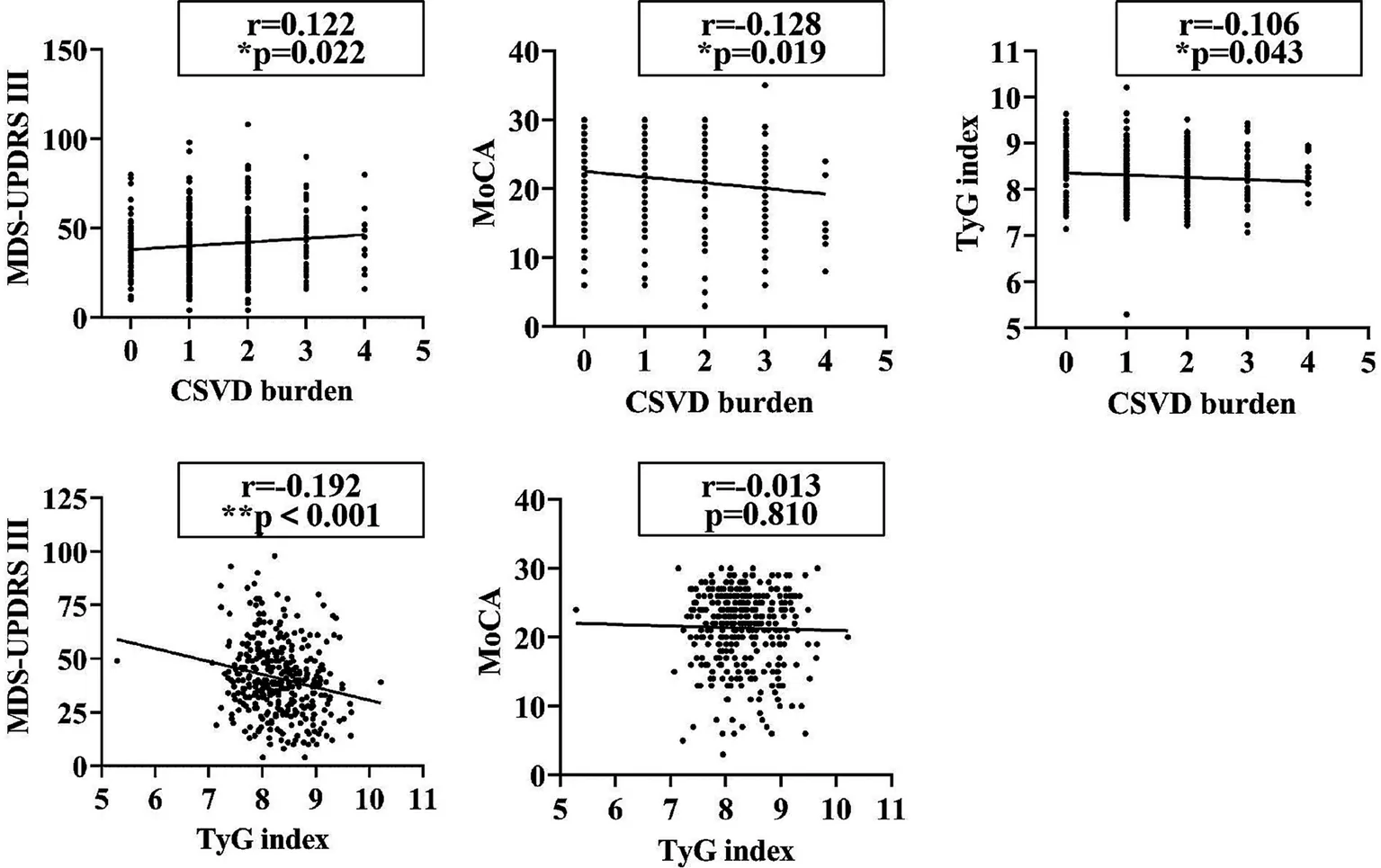
Spearman correlation analysis among CSVD burden, TyG index, MDS-UPDRS III, and MoCA scores. *p < 0.05, **p < 0.01. CSVD, cerebral small vessel disease burden; TyG index, triglyceride-glucose index; MDS-UPDRS III, Movement Disorder Society-Unified Parkinson’s Disease Rating Scale part III; MoCA, Montreal Cognitive Assessment.

Spearman correlation analysis evaluated the bivariate associations among CSVD burden, TyG index, motor and cognitive impairment in PD. As shown in Figure 2, CSVD burden was positively correlated with MDS-UPDRS III (*r* = 0.122, *p* = 0.022) and negatively correlated with MoCA (*r* = −0.128, *p* = 0.019). CSVD burden was also negatively correlated with the TyG index (*r* = −0.106, *p* = 0.043). TyG index was negatively correlated with MDS-UPDRS III (*r* = −0.192, *p* < 0.001), but exhibited no significant correlation with MoCA (*r* = −0.013, *p* = 0.810). These results supported further mediation analysis for motor function.

### 3.3 Mediation analysis for motor function

**Figure 3:**
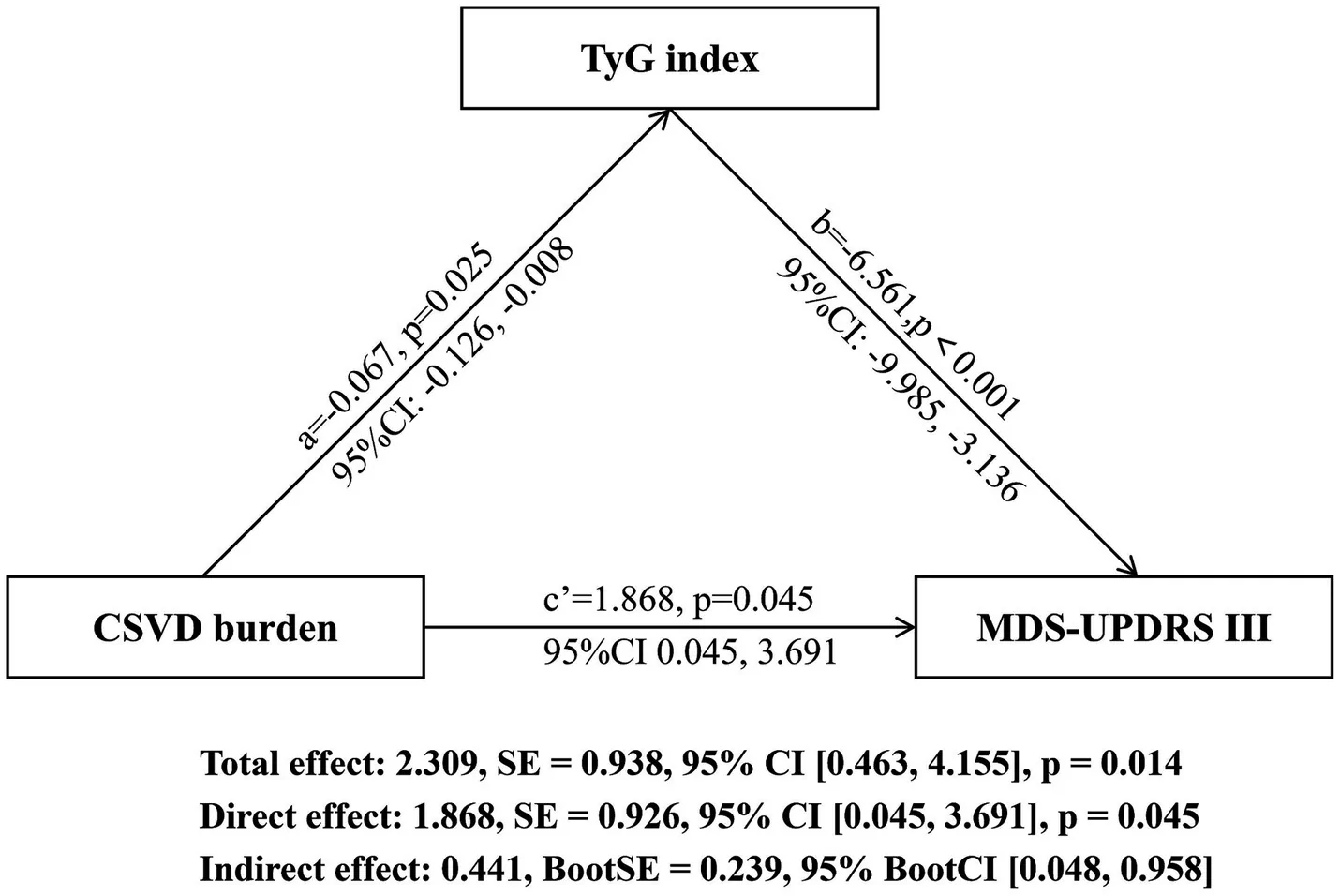
Mediation analysis of the TyG index in the relationship between CSVD burden and motor impairment. Values are unstandardized regression coefficients. The indirect effect was 0.441 [95% bootstrap CI (0.048, 0.958)], accounting for 19.1% of the total effect. All models were adjusted for age, sex, education, hypertension, diabetes, and LEDD. CSVD, cerebral small vessel disease burden; TyG index, triglyceride-glucose index; MDS-UPDRS III, Movement Disorder Society-Unified Parkinson’s Disease Rating Scale part III; CI, confidence intervals, LEDD, levodopa equivalent daily dosage.

We next examined whether the TyG index statistically mediated the association between CSVD burden and motor impairment in PD by PROCESS Model 4 with 5,000 bootstrap resamples. Age, sex, years of education, hypertension, diabetes, and LEDD were included as covariates. As summarized in Table 2 and Figure 3, the total effect of CSVD burden on MDS-UPDRS III was significant [*B* = 2.309, 95% CI (0.463, 4.155), *p* = 0.014]. CSVD burden was negatively correlated with TyG index [*a* = −0.067, 95%CI (−0.126, −0.008), *p* = 0.025]. The TyG index was negatively associated with MDS-UPDRS III [b = −6.561, 95%CI (−9.985, −3.136), *p* < 0.001]. The indirect effect was 0.441 (95%CI [0.048, 0.958]), which excluded zero, indicating statistically significant partial mediation. The direct effect remained significant after controlling for the TyG index [c’ = 1.868, 95%CI (0.045, 3.691), *p* = 0.045]. The proportion of the total effect mediated was 19.1%.

**Table 2:** Mediation analysis of TyG index on the relationship between CSVD burden and MDS-UPDRS III in PD.

Effects	B	SE	95% CI	*p*	Proportion mediated
Total effect	2.309	0.938	0.463, 4.155	0.014	/
Direct effect	1.868	0.926	0.045, 3.691	0.045	/
Indirect effect	0.441	0.239^a^	0.048, 0.958^b^	/	19.1%

^a^, Bootstrap SE; ^b^, Bootstrap 95% CI based on 5,000 resamples. All models adjusted for age, sex, years of education, hypertension, diabetes, and LEDD. Bootstrap CI that does not include zero indicates significant mediation. /, not applicable.

B, unstandardized coefficient; SE, standard error; CI, confidence intervals; LEDD, levodopa equivalent daily dosage.

### 3.4 Sensitivity analysis

To test the robustness and specificity of the mediation effect, we conducted several sensitivity analyses (Supplementary Table S2). When the TyG index was replaced by SII, SIRI, NLR and NHR, none of them showed a significant indirect effect (all bootstrap 95% CIs crossed zero). After dichotomizing CSVD burden into low and high groups, the indirect effect via the TyG index became non-significant [indirect effect = 0.657, 95% CI (−0.125, 1.744)]. Additionally adjusted for BMI, the significance remained [indirect effect = 0.397, 95% CI (0.019, 0.935); proportion mediated: 17.4%], supporting the robustness of the primary finding. When disease duration and H-Y staging were additionally included as covariates, the indirect effect remained significant [indirect effect = 0.288, 95% CI (0.019, 0.687)], suggesting that the mediating role of TyG index remains robust after adjusting for these key indicators of disease severity.

We further conducted mediation analysis using fasting triglycerides and glucose as individual mediators. In the forward direction, neither triglycerides [indirect effect = 0.227, 95% CI (−0.017, 0.603)] nor glucose [indirect effect = 0.050, 95% CI (−0.048, 0.260)] showed a statistically significant indirect effect. In the reverse direction, the indirect effects of TyG index [0.0012, 95% CI (−0.0001, 0.0028)], triglycerides [0.0009, 95% CI (−0.0002, 0.0026)] and glucose [0.0002, 95% CI (−0.0002, 0.0011)] were not statistically significant (Supplementary Table S2).

### 3.5 Secondary analysis for cognitive function

The formal mediation analysis for cognitive function was not pursued due to the lack of association between the TyG index and MoCA. For transparency, the mediation model was nonetheless applied, and no significant indirect effect was observed (indirect effect: 0.013, 95% CI: −0.048, 0.091) (Supplementary Table S3).

## 4 Discussion

In this retrospective cross-sectional study, we found that the TyG index significantly and partially statistically accounted for the association between CSVD burden and motor impairment in PD patients, accounting for approximately 19.1% of the total effect. This statistical mediation followed a negative-to-negative pathway: higher CSVD burden correlated with a lower TyG index, which in turn was associated with more severe motor dysfunction. The indirect effect remained robust across multiple sensitivity analyses and was specific to the TyG index. By contrast, the TyG index did not statistically account for the effect of CSVD burden on cognitive function, indicating domain specificity. To our knowledge, this is the first study to directly evaluate the mediating role of the TyG index in the CSVD-motor link in PD.

Our observation that the TyG index statistically mediated the association between CSVD and motor function aligned with accumulating evidence linking metabolic inflammation to PD severity (Ma et al., 2025a; Jiang et al., 2026; Li et al., 2026). Nonetheless, this directionality—higher CSVD burden associated with a lower TyG index, and a lower TyG index linked to worse motor function—appeared counterintuitive from a conventional metabolic perspective, where a higher TyG index typically signaled insulin resistance and poorer vascular health. Notably, the result was consistent with our previous finding that lower serum triglyceride levels were associated with more severe motor impairment in PD patients (Zhang et al., 2022). In general populations, an elevated TyG index is a risk factor for CSVD (Nam et al., 2020; Jung et al., 2022; Teng et al., 2022), yet in definite PD, the relationship may be reversed. This discrepancy may suggest that the metabolic phenotype of PD differs from that of non-PD populations. Disease-specific alterations in energy homeostasis, such as chronic malnutrition caused by dysphagia or a hypermetabolic state, may account for this pattern (Suttrup and Warnecke, 2016; Femat-Roldán et al., 2020).

Several possible mechanisms may explain the observed negative-to-negative pathway. First, as the disease progresses, PD patients frequently develop catabolic states such as malnutrition, sarcopenia, and weight loss, which are directly linked to more severe motor symptoms (Suttrup and Warnecke, 2016; Gui et al., 2025). Importantly, emerging evidence indicates that CSVD and its imaging markers are independently associated with an increased risk of malnutrition and sarcopenia (Li et al., 2025; Liu et al., 2025). Therefore, a higher CSVD burden may accelerate nutritional decline and muscle loss in PD patients, perpetuating a chronic state of depletion that lowers triglyceride and blood glucose levels and ultimately reduces the TyG index.

Second, central and peripheral metabolic perturbations may exert additive or synergistic effects. CSVD may simultaneously disrupt central insulin signaling through chronic hypoperfusion (Deery et al., 2024) and promote peripheral catabolism via systemic inflammation or neuroendocrine dysregulation (Liu et al., 2025), resulting in both central metabolic dysfunction and a reduced peripheral TyG index. Consequently, a lower TyG index diminishes the supply of energy substrates to the brain, further compromising neuronal energy availability in the already vulnerable nigrostriatal pathway and thereby worsening motor impairment of PD. However, we acknowledge that our study lacked direct measures of nutritional status, sarcopenia, or caloric intake. An alternative interpretation is that the observed association between CSVD and lower TyG index may reflect the recognized link between age-related vascular burden and declining nutritional biomarkers, rather than a PD-specific metabolic shift (Liu et al., 2025). We also acknowledge that alternative directional models remain equally plausible. For instance, more severe motor impairment could lead to reduced physical activity, nutritional decline, and metabolic changes reflected in a lower TyG index, which could also produce the observed cross-sectional associations. To explore these possibilities, we conducted two additional analyses. Specifically, we decomposed the TyG index; neither fasting triglycerides nor fasting glucose showed a significant indirect effect, suggesting the composite TyG index captures synergistic associations among metabolic indicators, rather than isolated changes in a single metabolite. Additionally, a reverse mediation model (MDS-UPDRS III → M → CSVD burden) yielded a non-significant indirect effect, suggesting this reverse pathway lacked statistical support. Nevertheless, cross-sectional data cannot definitively distinguish between competing directional models, and our findings should be viewed as hypothesis-generating for subsequent longitudinal validation studies.

Third, the effects of dopaminergic medications are unlikely to be the sole driver, as the mediation remained significant after adjusting for LEDD. However, these proposed mechanisms remain speculative and require confirmation through future longitudinal or interventional studies.

The specificity of the TyG index as a mediator was supported by sensitivity analyses incorporating four systemic inflammation indicators (SII, SIRI, NLR, and NHR), none of which demonstrated a significant indirect effect. This finding suggested that the observed mediation was specific to the metabolic-insulin resistance pathway captured by the TyG index, rather than to generalized inflammation. When CSVD burden was dichotomized, the indirect effect lost statistical significance. Notably, the point estimate (0.657) was larger than in the continuous model (0.441), but the confidence interval was substantially wider. This is consistent with reduced statistical power from dichotomization and loss of gradient information, rather than the absence of an effect. It supports a dose–response interpretation over a threshold effect, but also warns that the mediation may not withstand coarse categorization of the exposure. Additional adjustment for BMI, disease duration, and H-Y staging did not materially alter the indirect effect, further reinforcing the robustness of these results. The correlation between TyG and BMI in our cohort was moderate (*r* = 0.312, *p* < 0.001), suggesting that adjustment for BMI does not represent overadjustment.

In contrast to previous studies that reported a moderate to strong negative correlation between the TyG index and MMSE or MoCA scores in PD (Cao et al., 2025; Cheng et al., 2025), we found neither a significant bivariate correlation nor a mediation effect of TyG index on the CSVD-cognition link. This discrepancy may reflect genuine metabolic heterogeneity in PD. The strong association observed in prior cohort studies may have been driven by a higher proportion of the hyperinsulinemic phenotype [elevated TyG index; mean level was 8.35 ± 0.47 (Cao et al., 2025)]. Our cohort, by contrast, likely contained a larger proportion of individuals with a catabolic or exhaustion phenotype (low TyG index; mean level was 8.29 ± 0.56). This predominantly catabolic profile may attenuate the association between the TyG index and cognitive function, even though the index still captures relative differences in insulin resistance-related metabolic status within this population. In addition, a recent machine learning study suggested that the TyG index is linearly and negatively correlated with specific cognitive domains such as memory and orientation, whereas its relationship with visuospatial function and attention followed an inverted U-shaped pattern (Liang et al., 2026). However, the simple bivariate correlation analyses used in our study may not adequately capture such complex patterns of variation. Furthermore, the cross-sectional design may be less sensitive to capturing TyG’s effect on cognitive decline.

Our study demonstrated a potential statistical mediating role of the TyG index in the association between CSVD and motor function in PD, suggesting a hypothesis-generating target for CSVD-related motor impairment that warrants prospective investigation. Nevertheless, several limitations should be acknowledged. First, the retrospective cross-sectional design precludes causal inference. We acknowledge that alternative directional models remain equally plausible. While a reverse mediation analysis yielded a non-significant indirect effect, this does not prove the forward direction. Prospective longitudinal studies are required to validate these findings. Second, detailed metabolic and nutritional data were unavailable in this retrospective cohort. We lacked information on statin use and dietary intake, both of which directly influence triglyceride levels and the TyG index. Additionally, objective nutritional indicators, such as albumin and prealbumin levels, which could further support the catabolic hypothesis, along with physical activity level data and PD motor subtype, were unavailable. Moreover, the absence of PD motor subtype data precluded examination of phenotype-specific mediation effects, particularly given the reported association between CSVD and the postural instability and gait difficulty subtype (Ge et al., 2025). Consequently, the potential confounding effects of these unmeasured variables on the observed associations cannot be entirely ruled out. Third, the MoCA is a global cognitive screening tool that may lack sensitivity to subtle deficits in specific cognitive domains. Fourth, this study relied on routine clinical, laboratory and imaging data not originally gathered for research purposes. Such retrospective real-world data inherently carry limitations, including potential diagnostic misclassification, unmeasured confounding, and minor temporal variations in clinical assessments. Although incomplete records were excluded, biases inherent to data quality could not be entirely eliminated. Finally, whether the findings of our study extend to other ethnic populations and clinical settings warrant validation through independent studies.

## 5 Conclusion

In conclusion, our study found the TyG index statistically and partially accounted for the association between CSVD burden and motor impairment in PD, with a negative-to-negative pathway. As a simple and inexpensive biomarker, the TyG index may reflect a modifiable metabolic pathway associated with CSVD-related motor impairment, offering a hypothesis-generating target for future interventional studies. Prospective longitudinal studies and interventional trials are warranted to confirm these findings and to explore whether targeting metabolic pathways could improve CSVD-related motor impairment.

## Data Availability

The raw data supporting the conclusions of this article will be made available by the authors, without undue reservation.
