# Supplementary material for: Triglyceride-glucose index is associated with cerebral small vessel disease-related motor impairment in Parkinson’s disease: a mediation analysis
Source: Front Aging Neurosci. 2026 Aug 10;18:1902923. doi: 10.3389/fnagi.2026.1902923 (PMC13500512; doi:10.3389/fnagi.2026.1902923)
Supplement: Supplementary file 1 [file Data_Sheet_1.DOCX]

**Supplementary Table S1** Frequencies and proportions of CSVD imaging markers

| **CSVD imaging markers** | **Category** | **N (%)** |
| --- | --- | --- |
| Periventricular hyperintensities | Fazekas 0 | 91 (25.14%) |
|  | Fazekas 1 | 127 (35.08%) |
|  | Fazekas 2 | 81 (22.38%) |
|  | Fazekas 3 | 63 (17.40%) |
| Deep white matter hyperintensities | Fazekas 0 | 87 (24.03%) |
|  | Fazekas 1 | 186 (51.38%) |
|  | Fazekas 2 | 75 (20.72%) |
|  | Fazekas 3 | 14 (3.87%) |
| Enlarged perivascular spaces in the basal ganglia | Grade 0 | 69 (19.06%) |
|  | Grade 1 | 221 (61.05%) |
|  | Grade 2 | 54 (14.92%) |
|  | Grade 3 | 13 (3.59%) |
|  | Grade 4 | 5 (1.38%) |
| Lacunes | Absent | 117 (32.32%) |
|  | Present | 245 (67.68%) |
| Microbleeds | Absent | 148 (40.88%) |
|  | Present | 214 (59.12%) |

**Supplementary Table S2** Sensitivity analyses for the mediation effect of TyG index.

| **Sensitivity analysis** | **Indirect effect** | **Boot SE** | **Boot 95% CI** | **Conclusion** |
| --- | --- | --- | --- | --- |
| **Primary analysis ((TyG index as mediator))** | 0.441 | 0.239 | 0.048, 0.958 | Significant |
| **Alternative mediators** |  |  |  |  |
| SII | 0.012 | 0.085 | -0.032, 0.277 | Not significant |
| SIRI | -0.177 | 0.169 | -0.530, 0.134 | Not significant |
| NLR | 0.024 | 0.067 | -0.093, 0.171 | Not significant |
| NHR | -0.008 | 0.069 | -0.134, 0.164 | Not significant |
| **CSVD burden dichotomized (high vs. low)** | 0.657 | 0.474 | -0.125, 1.744 | Not significant |
| **Additional adjustment for BMI** | 0.397 | 0.234 | 0.019, 0.935 | Significant  (proportion mediated: 17.4%) |
| **Additional adjustment for disease duration and H-Y staging** | 0.288 | 0.176 | 0.019,0.687 | Significant  (proportion mediated: 15.0%) |
| **Decomposition of TyG index (forward: CSVD → M → UPDRS-III)** |  |  |  |  |
| Triglycerides | 0.227 | 0.166 | -0.017, 0.603 | Not significant |
| Glucose | 0.050 | 0.078 | -0.048, 0.260 | Not significant |
| **Decomposition of TyG index (reverse: UPDRS-III → M → CSVD)** |  |  |  |  |
| TyG index | 0.0012 | 0.0007 | -0.0001, 0.0028 | Not significant |
| Triglycerides | 0.0009 | 0.0007 | -0.0002, 0.0026 | Not significant |
| Glucose | 0.0002 | 0.0003 | -0.0002, 0.0011 | Not significant |

Abbreviations: CSVD, cerebral small vessel disease burden; SII, neutrophils×platelets/lymphocytes; SIRI, neutrophils×monocytes/lymphocytes; NLR, neutrophil-to-lymphocyte ratio; NHR, neutrophil-to-HDL-C ratio; BMI, body mass index; SE, standard error; CI, confidence intervals.

**Supplementary Table S3** Mediation analysis of TyG index on the relationship between CSVD burden and MoCA

| **Effect** | **B** | **SE** | **95% CI** | **p** |
| --- | --- | --- | --- | --- |
| Total effect | -0.576 | 0.285 | -1.136, -0.016 | 0.044 |
| Direct effect | -0.589 | 0.286 | -1.152, -0.026 | 0.040 |
| Indirect effect | 0.013 | 0.034^a^ | -0.048, 0.091^b^ | / |

^a^, Bootstrap SE; ^b^, Bootstrap 95% CI based on 5,000 resamples. All models adjusted for age, sex, years of education, hypertension, diabetes, and LEDD. Bootstrap CI that does not include zero indicates significant mediation. /, not applicable.

Abbreviations: B, unstandardized coefficient; SE, standard error; CI, confidence intervals; LEDD, levodopa equivalent daily dosage.
